# Identification of distinct impacts of CovS inactivation on the transcriptome of acapsular group A streptococci

**DOI:** 10.1128/msystems.00227-23

**Published:** 2023-06-26

**Authors:** Sruti DebRoy, William C. Shropshire, Luis Vega, Chau Tran, Nicola Horstmann, Piyali Mukherjee, Selvalakshmi Selvaraj-Anand, Truc T. Tran, Jordan Bremer, Marc Gohel, Cesar A. Arias, Anthony R. Flores, Samuel A. Shelburne

**Affiliations:** 1 Department of Infectious Diseases Infection Control and Employee Health, University of Texas MD Anderson Cancer Center, Houston, Texas, USA; 2 Division of Infectious Diseases and Department of Pediatrics, McGovern Medical School at UTHealth Houston and Children’s Memorial Hermann Hospital, Houston, Texas, USA; 3 School of Health Professions, The University of Texas MD Anderson Cancer Center, Houston, Texas, USA; 4 Center for Infectious Diseases, Houston Methodist Research Institute, Houston, Texas, USA; 5 Division of Infectious Diseases, Department of Medicine, Houston Methodist Hospital, Houston, Texas, USA; 6 Department of Genomic Medicine, The University of Texas MD Anderson Cancer Center, Houston, Texas, USA; California State University, Stanislaus, Turlock, California, USA

**Keywords:** Streptococcus, CovRS, acapsular

## Abstract

Group A streptococcal (GAS) strains causing severe, invasive infections often have mutations in the control of virulence two-component regulatory system (CovRS) which represses capsule production, and high-level capsule production is considered critical to the GAS hypervirulent phenotype. Additionally, based on studies in *emm1* GAS, hyperencapsulation is thought to limit transmission of CovRS-mutated strains by reducing GAS adherence to mucosal surfaces. It has recently been identified that about 30% of invasive GAS strains lacks capsule, but there are limited data regarding the impact of CovS inactivation in such acapsular strains. Using publicly available complete genomes (*n* = 2,455) of invasive GAS strains, we identified similar rates of CovRS inactivation and limited evidence for transmission of CovRS-mutated isolates for both encapsulated and acapsular *emm* types. Relative to encapsulated GAS, CovS transcriptomes of the prevalent acapsular *emm* types *emm28*, *emm87*, and *emm89* revealed unique impacts such as increased transcript levels of genes in the *emm*/mga region along with decreased transcript levels of pilus operon-encoding genes and the streptokinase-encoding gene *ska*. CovS inactivation in *emm87* and *emm89* strains, but not *emm28*, increased GAS survival in human blood. Moreover, CovS inactivation in acapsular GAS reduced adherence to host epithelial cells. These data suggest that the hypervirulence induced by CovS inactivation in acapsular GAS follows distinct pathways from the better studied encapsulated strains and that factors other than hyperencapsulation may account for the lack of transmission of CovRS-mutated strains.

**IMPORTANCE:**

Devastating infections due to group A streptococci (GAS) tend to occur sporadically and are often caused by strains that contain mutations in the control of virulence regulatory system (CovRS). In well-studied *emm1* GAS, the increased production of capsule induced by CovRS mutation is considered key to both hypervirulence and limited transmissibility by interfering with proteins that mediate attachment to eukaryotic cells. Herein, we show that the rates of covRS mutations and genetic clustering of CovRS-mutated isolates are independent of capsule status. Moreover, we found that CovS inactivation in multiple acapsular GAS *emm* types results in dramatically altered transcript levels of a diverse array of cell-surface protein-encoding genes and a unique transcriptome relative to encapsulated GAS. These data provide new insights into how a major human pathogen achieves hypervirulence and indicate that factors other than hyperencapsulation likely account for the sporadic nature of the severe GAS disease.

## INTRODUCTION

Group A *Streptococcus* (GAS) is among the leading causes of invasive bacterial disease in humans and has long served as a model organism for investigating serious bacterial infections (1). GAS causes a wide array of infections, ranging from uncomplicated pharyngitis and cellulitis to life-threatening diseases such as necrotizing fasciitis and streptococcal toxic shock syndrome (2). In part, this variation in disease is due to inactivating mutations in the control of virulence sensor kinase (CovS) which impacts the phosphorylation state and, hence activity, of its cognate control of virulence response regulator (CovR) (3 - 5). Phosphorylated CovR (CovR~P) primarily represses virulence factor production, and inactivation of CovS decreases CovR~P level leading to increased expression of virulence factor-encoding genes and thereby hypervirulent GAS strains (3, 6 - 8).

The major typing scheme for GAS is based on the hypervariable 5′ sequence of the *emm* gene, which encodes the key GAS cell surface virulence factor, *M* protein (9). The vast majority of work on the control of virulence two-component regulatory system (CovRS) system has been in the *emm1* pandemic clone M1T1 in which the antiphagocytic *M* protein and hyaluronic acid capsule have been shown to be necessary for the emergence of hypervirulent GAS (3, 10 - 16). CovR~P directly represses the hyaluronic acid capsule operon (3, 17), and the hyperproduction of capsule in the M1T1 background induced by CovS inactivation inhibits GAS adherence to epithelial cells, which, in turn, is thought to limit transmissibility of CovS-inactivated strains (13, 18). Indeed, multiple studies have found that CovS-inactivated strains generally tend to cause only one or very limited numbers of infections (19 - 21).

The GAS hyaluronic acid capsule has long been considered a key virulence factor because of its critical role in inhibiting phagocytosis, and CovS inactivation improves M1T1 GAS survival during interaction with neutrophils, a process considered critical to the emergence of CovS-mutated strains (10, 22 - 26). However, whole-genome sequencing has recently identified GAS *emm* types that either lack the *hasABC* operon that encodes capsule synthesis proteins (*emm4, emm22*) or have conserved mutations in *hasA* that abolish capsule production (*emm28*, *emm87*) (27 - 31). Similarly, unencapsulated *emm89* strains have been replacing encapsulated *emm89* isolates over the past decade in numerous countries (32 - 34). GAS strains are divided into *emm* patterns (A–C, D, and E) based on *emm* region content, and all currently known capsule-negative GAS *emm* types are pattern E (9, 35). Together, these pattern E, unencapsulated strains accounted for ~33% of invasive GAS disease in the most recent Centers for Disease Control and Prevention (CDC) invasive GAS surveillance report (36).

We previously identified the emergence of CovS inactivation during infection in an *emm4* GAS strain and found that the acapsular, CovS-inactivated strain was hypervirulent (37). Given the critical role of capsule in the emergence of CovS-inactivated strains in the M1T1 background, it was somewhat surprising to observe that CovS-inactivated strains could be identified in acapsular *emm4* strains (29, 37). Inasmuch as *emm4* strains are relatively rare causes of invasive GAS infections, we sought to characterize the impact of CovS inactivation in other acapsular *emm* types. We found that CovS inactivation in *emm28*, *emm87*, and *emm89* strains resulted in a unique transcriptional impact leading to upregulation of *emm* and the multigene activator-encoding gene (*mga*) along with downregulation of the pilus-encoding operon and the gene-encoding streptokinase (SKA). In addition, CovS inactivation reduced the adherence of acapsular GAS strains to tonsillar epithelial cells, an observation that could potentially explain the rarity of genetic clusters of CovRS-mutated, acapsular isolates. These data provide a new mechanism by which a major human pathogen achieves hypervirulence and suggest that factors other than hyperencapsulation may limit transmission of hypervirulent GAS.

## RESULTS

### Rate of *covRS* polymorphisms predicted to alter CovRS function is similar in encapsulated and acapsular invasive GAS strains

To compare the rates of CovRS mutations in acapsular vs. encapsulated GAS, we downloaded 2,455 publicly available genomes from National Center for Biotechnology Information (NCBI) of invasive GAS strains collected by the CDC using active surveillance (accessed 4 July 2022) (36). We then analyzed the *covRS* region and identified genetic polymorphisms in terms of predicted impact (e.g., synonymous, nonsynonymous, nonsense, frameshift, etc.). The most commonly identified *covR* polymorphisms that impacted protein composition were missense (97%) and nonsense (3%) mutations, whereas for *covS*, 58% were nonsynonymous polymorphisms, 24% were frameshifts, and 18% were nonsense mutations. There was no significant difference in the types of polymorphisms observed between encapsulated and acapsular strains (*P-*value = 0.9 by χ^2^ analysis). Among all *emm* types with at least 20 isolates, the highest rates of predicted functionally significant *covRS* polymorphisms were observed for *emm28* (35.7%). The rates of *covRS* polymorphisms predicted to alter function in the five most common encapsulated *emm* types (*emm1*, *emm49*, *emm12*, *emm3*, and *emm82*) vs. the five most common acapsular *emm* types (*emm89*, *emm28*, *emm4*, *emm77*, and *emm87*) were not significantly different (*P* = 0.8 by Student’s *t*-test). Similarly, no significant difference was observed when comparing the rates of *covRS* polymorphisms in encapsulated vs. acapsular strains (18% vs. 23%, *P*-value = 0.43 by χ^2^ analysis). These data indicate that the presence/absence of capsule does not significantly impact the rates of *covRS* polymorphisms among invasive GAS.

### Genetic clustering of *CovRS-*mutated, invasive strains is rare in both encapsulated and acapsular *emm* types

**Fig 1:**
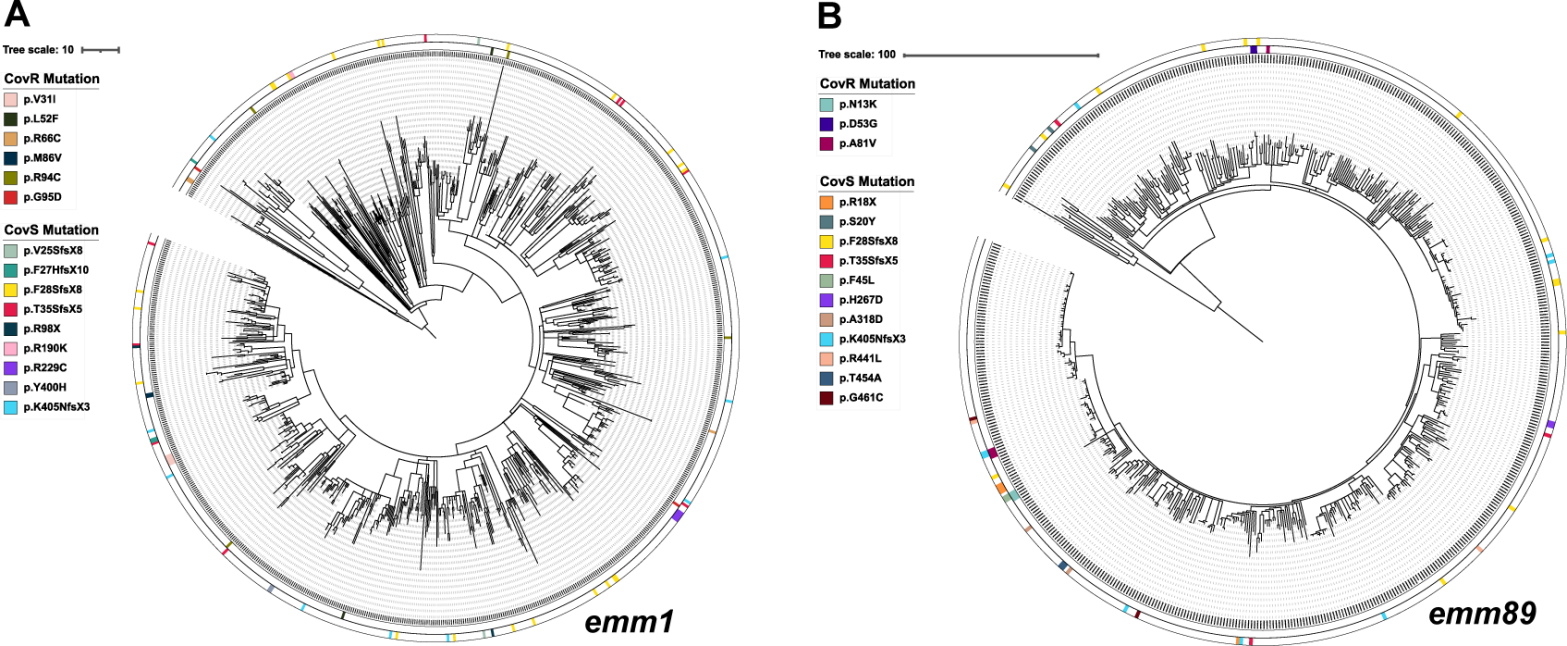
Occurrence and genetic clustering of *covRS* mutations in encapsulated *emm1* and acapsular *emm89* invasive strains. A recombination-free, core genome alignment inferred maximum-likelihood phylogenetic tree was created from alignment of (**A**) 857 *emm1* strains and (**B**) 562 *emm89* strains. The occurrence of mutations in *covR* and *covS* is indicated on the inner and outer circles, respectively, and color coordinated for each specific mutation with corresponding amino acid change as detailed in the key. For frameshift mutations, the first altered amino acid is indicated followed by the distance to the premature stop (e.g., V25SfsX8 implies a frameshift that alters residues from amino acid 25 and results in a premature stop eight residues downstream).

It has been theorized that the hyperproduction of capsule due to CovS inactivation hinders transmission of hypervirulent, encapsulated GAS (13). Thus, one might hypothesize that CovS-inactivated, acapsular GAS would not have such a transmission defect and could cause outbreaks. Indeed, we have previously described a family cluster of invasive, CovS-inactivated, acapsular *emm87* GAS (38). We overlaid our *covRS* polymorphism data onto the core genome phylogenetic trees of common encapsulated (*emm1*, *emm3*, and *emm12*) as well as acapsular (*emm28*, *emm87*, and *emm89*) *emm* types to search for instances where strains with the same *covRS* polymorphism clustered together (Fig. 1). For a given *emm* type, the vast majority of strains with a specific *covRS* polymorphism occurred only a single time (259/319, 81%) (Table S1). There was no significant difference between encapsulated and acapsular strains in the rates at which a specific polymorphism occurred more than once in the same *emm* type (Fisher’s exact test *P*-value = 0.41). For both encapsulated and acapsular GAS, the only instances of genetic clustering of three or more strains occurred for isolates with missense mutations (i.e., not frameshifts or nonsense) (Fig. 1; Fig. S1). Conversely, strains with frameshifts or nonsense mutations, which almost always occurred in CovS rather than CovR, were genetically heterogenous with the largest cluster being two strains (Fig. 1; Fig. S1). For each specific *covRS* polymorphism within a given *emm* type, genetic clustering rarely occurred (10% of cases) and was not significantly different between encapsulated (10%) and acapsular strains (10%), *P*-value = 1.0 by Fisher’s exact test (Table S1). Taken together, we conclude that these data suggest no significant impact of capsule presence on the capacity of *covRS-*mutated strains to cause outbreaks.

### Characterization of CovS-inactivated, acapsular *emm28*, *emm87*, and *emm89* strains

**Fig 2:**
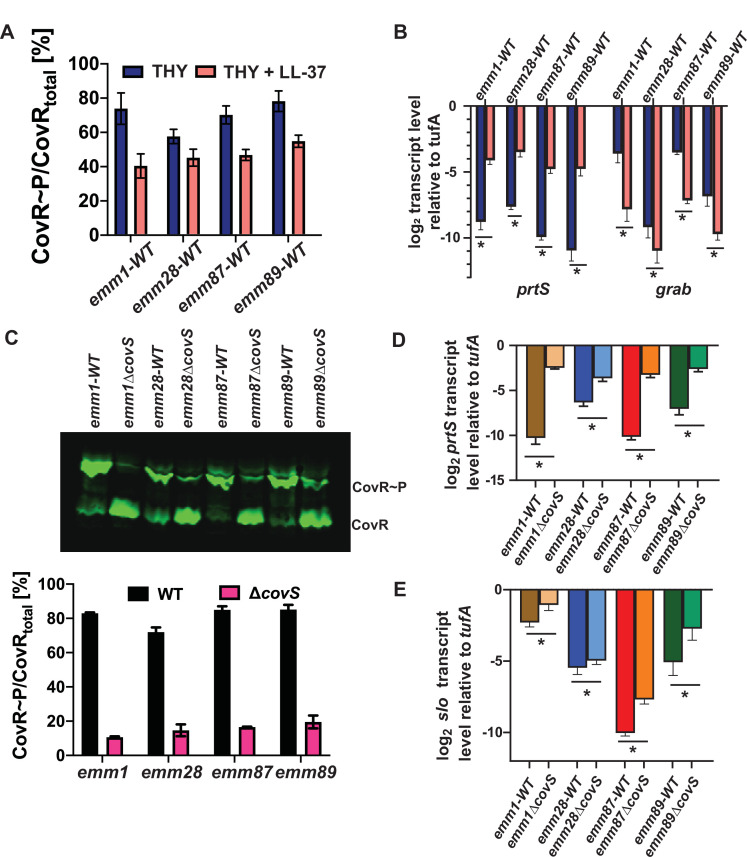
Impact of CovS inactivation on cellular CovR~P levels and transcripts of CovRS-responsive genes. (**A**) Graphical representation of representative Phos-Tag Western blot of phosphorylated CovR (CovR~P) levels and (**B**) Taqman qRT-PCR analysis of CovR~P responsive gene transcripts in *emm1*, *emm28*, *emm87,* and *emm89* wild-type strains treated with LL37. (**C**) PhosTag Western blot (*n* = 2) and graphical representation of CovR~P levels and (**D, E**) transcript levels of *prtS* and *slo* in wild-type and CovS-inactivated *emm1*, *emm28*, *emm87,* and *emm89* strains. Recombinant-purified CovR/CovR~P was used as controls. Error bars indicate standard deviation. For Taqman qRT-PCR analysis, data are means ± standard deviations of two biological replicates, with two technical replicates, done on two separate days.

To gain insights into how CovS functions in acapsular GAS, we chose to study *emm*28, *emm*87, and *emm*89 strains because each is a common GAS *emm* type and the *emm* types are genetically diverse relative to each other (39). Each of the chosen wild-type strains, TSPY902 (herein called *emm28-*WT), TSPY1057 (herein called *emm87-*WT), and MSPY1 (*emm89-*WT), has been fully sequenced and is wild type for all major transcriptional regulators (40). CovS mediates the response of *emm*1 GAS to the immune antimicrobial peptide LL37 by lowering CovR~P levels and, thus, relieving CovR repression of critical virulence factor-encoding genes (41, 42). We confirmed the activity of CovS in our wild-type acapsular strains through exposure to LL-37 which resulted in decreased levels of CovR~P (Fig. 2A), increased transcript levels of the CovR~P-repressed gene *prtS*, and decreased transcript levels of the CovR~P activated gene *grab* (Fig. 2B). *PrtS* encodes an IL-8 degrading protease, whereas *grab* encodes an alpha2 macroglobulin-like binding protein. The direction of these transcript level changes (i.e., increase for *prtS* and decrease for *grab* following LL-37 exposure) was the same as observed for the well-characterized M1T1 (*emm*1) strain MGAS2221 (herein called *emm1*) (Fig. 2A and B).

We used non-polar insertional inactivation to remove the entire *covS* open reading frame from the parental acapsular strains (43) (Table 1). For all three *emm* types, CovS inactivation did not significantly impact growth in nutrient-rich media nor phenotype on blood agar. Consistent with observations in *emm*1 strains, CovS inactivation in the acapsular strains significantly reduced the amount of CovR~P (Fig. 2C). CovR~P levels were similar for all three CovS-inactivated strains. However, the amount of CovR~P in the wild-type *emm*28 strain was lower compared to the wild-type *emm*87 and *emm*89 strains, perhaps due to a conserved CovS E226G polymorphism present in all sequenced *emm*28 strains (44). As observed in *emm1* strains, inactivation of CovS in the acapsular GAS strains resulted in increased transcript levels of *prtS* and *slo* genes (Fig. 2D and E), which encode the pore-forming toxin streptolysin O (Slo).

**TABLE 1:** Strains used in this work

Strain or plasmid	Description	Reference
MGAS2221	Invasive clinical isolate, serotype *emm1*, CovRS wild type	(11)
2221Δ*covS*	Isogenic *covS* mutant	(12)
2221∆*covR*	Isogenic *covR* mutant	(12)
TSPY902	Clinical isolate, serotype *emm28*, CovRS wild type	This study
TSPY902Δ*covS*	Isogenic *covS* mutant	This study
TSPY902∆*covR*	Isogenic *covR* mutant	This study
TSPY1057	Clinical isolate, serotype *emm87*, CovRS wild-type	(38)
TSPY1057Δ*covS*	Isogenic *covS* mutant	This study
TSPY1057∆*covR*	Isogenic *covR* mutant	This study
MSPY1	Clinical isolate, serotype *emm89*, CovRS wild type	(45)
MSPY1Δ*covS*	Isogenic *covS* mutant	This study
MSPY1∆*covR*	Isogenic *covR* mutant	This study

**Fig 3:**
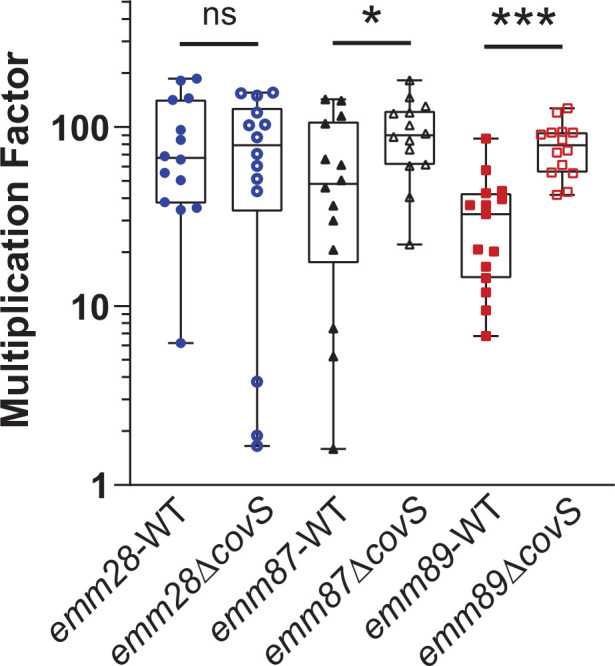
Impact of CovS inactivation on the ability of different acapsular GAS strains to multiply in human blood. Survival of wild-type (closed symbols) and CovS-inactivated (open symbols) *emm1*, *emm28*, *emm87,* and *emm89* strains in *ex vivo* human blood. Error bars represent standard deviations for GAS strains assayed in triplicate using four independent donors. Statistical significance calculated using Mann-Whitney *U* test is indicated by * (*P* < 0.05) and ** (*P* < 0.0005).

The impact of CovS inactivation on GAS virulence is heterogenous with hypervirulence consistently observed for *emm*1 strains (6, 11, 46, 47), whereas studies in non-*emm1* GAS have shown hypervirulence, hypovirulence, or no virulence impact depending on the strain and virulence assay used (18, 22, 48, 49). We analyzed the virulence of the *emm*28, *emm*87, and *emm*89 strains and their respective CovS-inactivated mutants in an *ex vivo* human blood model (6, 50 - 52). Similar to *emm*1 strains (6, 41), CovS inactivation in the *emm*87 and *emm*89 background survived better in blood compared to their wild-type counterparts (Fig. 3). However, we did not observe a statistically significant difference following CovS inactivation in the *emm*28 strain because the multiplication value for the *emm*28-wt strain was higher than *emm*87-wt or *emm*-89 wt, perhaps due to the lower baseline CovR~P observed in the *emm*28-wt strain.

### Characterization of the CovS transcriptome in acapsular GAS

**Fig 4:**
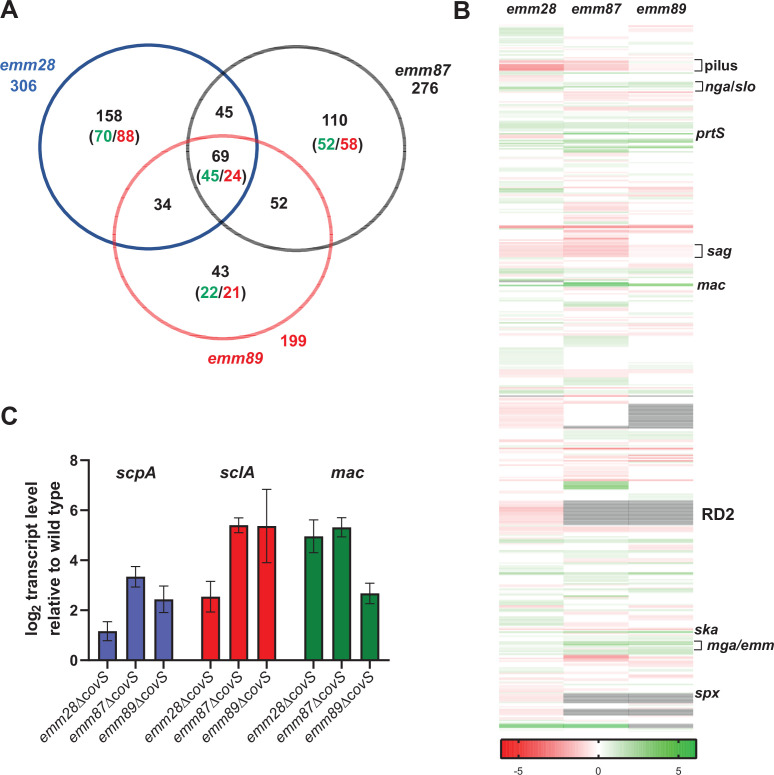
The CovS transcriptomes of acapsular *emm28*, *emm87,* and *emm89* GAS strains. (**A**) Venn diagram and (**B**) heat map representation of genes significantly differentially expressed upon CovS inactivation. Genes upregulated in ∆*covS* mutants, that is, CovR~P-repressed are indicated in green, and genes downregulated in ∆*covS* mutants, that is, CovR~P-activated are indicated in red. White indicates differential gene expression that was not statistically significant, and gray lines indicate the absence of a specific gene in a strain. (**C**) Taqman qRT-PCR analysis of select transcripts. Data shown represent means ± standard deviations of two biological replicates, with two technical replicates, done on two separate days.

To gain further understanding into the function of CovS in acapsular GAS, we performed RNA-seq on wild-type and CovS-inactivated strains grown to mid-exponential phase for the three chosen acapsular GAS *emm* types. Principal component analysis for each set of wild-type and CovS-inactivated strains showed clustering of samples by specific strains (i.e., CovS inactivated vs. wild type) (Fig. S2). Significant differential gene expression (DGE) was defined as a difference of 1.5-fold between the wild-type and CovS-inactivated strains with an adjusted Wald Test *P*-value ≤0.05. We identified 306, 276, and 199 significantly differentially expressed genes in CovS-inactivated mutants compared to wild-type *emm28*, *emm87,* and *emm89* strains, respectively (Fig. 4A; Table S2). There were 69 genes with significant DGE in all three *emm* types and an additional 131 having significant DGE in two of the three *emm* types (Fig. 4A). Of these 69 core genes, transcript levels were significantly increased for 45 genes (65%) and decreased for 24 genes (35%) following CovS inactivation, consistent with CovR primarily functioning as a repressor (3, 17). Genes whose transcript levels increased following CovS inactivation in all three *emm* types included *prtS* (53), *mac-1*, which encodes an immunoglobulin degrading enzyme (54), *sagA,* which is the first gene in the streptolysin S-encoding operon (55), *emm*, and the transcriptional regulator *spxA2* (56, 57). Genes whose transcript levels consistently decreased following CovS inactivation included *speC*, which encodes a superantigen (58), the cyclic AMP factor encoded by *cfa* (59), and *ska*, which encodes the plasminogen-activating enzyme streptokinase (60). Genes whose transcript levels were increased in two of three *emm* types included *mga* (61), *rivR*, which encodes a RofA-like transcriptional regulator (61), and genes in the streptolysin O-encoding operon (*nga/slo*) (62). Conversely, genes whose transcript levels were decreased in two of three *emm* types following CovS inactivation included those in the pilus (63) and the remainder of the streptolysin S-encoding operon. Finally, we observed large tracts of downregulated genes uniquely present in the *emm*28 strain which included genes in the prophage region as well as the so-called region of difference 2 (RD2) which shares significant homology with GBS (Fig. 4B) (64). We used qRT-PCR to confirm the RNA-seq findings for three genes that are upregulated upon CovS inactivation and found consistent results (Fig. 4C).

### Comparison of the acapsular GAS transcriptome with genes bound by CovR in *emm*1 GAS

**Fig 5:**
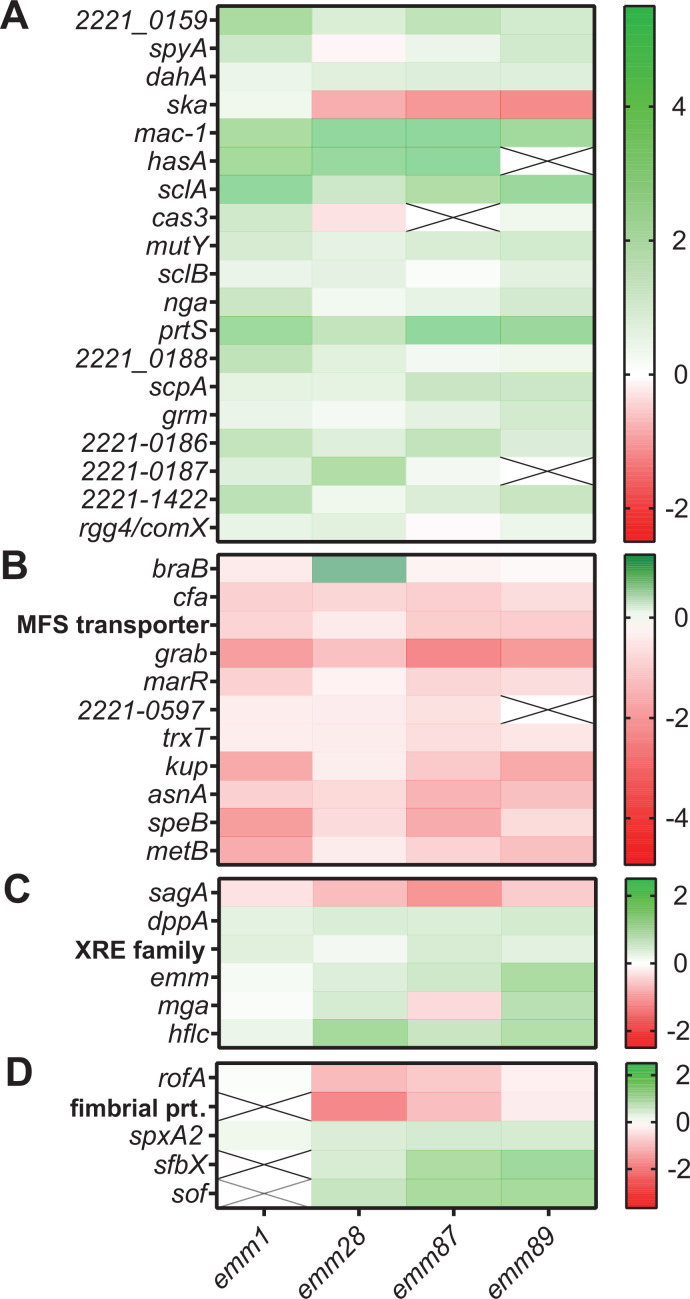
Comparison of the acapsular GAS transcriptome with genes bound by CovR in *emm*1 GAS. Heat maps comparing the expression levels of genes in CovS-inactivated *emm1* and acapsular strains identified as (**A**) directly CovR~P repressed and (**B**) directly CovR~P activated in *emm1* strain MGAS2221. (**C**) Genes that are directly bound by CovR~P in MGAS2221 but are transcriptionally altered only in acapsular strains upon CovS inactivation. (**D**) Genes that are either absent or not bound by CovR~P in MGAS2221 but altered transcriptionally in ∆*covS* mutants of acapsular GAS strains. Each column indicates transcript levels in ∆*covS* mutant relative to wild type for respective strain. Genes upregulated upon CovS inactivation are indicated in green, and genes downregulated upon CovS inactivation are indicated in red. A cross indicates the absence of a gene in that *emm* type.

To gain insight into potential mechanisms by which CovS impacts acapsular GAS gene expression, we compared our acapsular transcriptome analyses to our previously published CovR chromatin immunoprecipitation and DNA sequencing (ChIP-Seq) results and CovS transcriptome data from the *emm1* strain MGAS2221 (6, 17). The largest category of genes directly regulated by CovR in MGAS2221 is CovR~P repressed (i.e., increased transcript levels following CovS inactivation), and we observed very similar transcript level patterns for such genes in each of the acapsular strains (Fig. 5A). These included well-described CovR directly regulated virulence factor-encoding genes as *prtS*, *sclA* which encodes a cell-surface collagen-binding protein (65), and *mac-1*. Transcript levels of the *hasABC* operon, which is present in *emm*87 and *emm*28 strains but does not produce capsule due to point mutations (66), were elevated following CovS inactivation similar to what has been observed for *emm*1 (6, 12, 13, 67). The main exception to this concordance was *ska*, whose transcript level is increased by CovS inactivation in *emm1* GAS (6, 11, 67) but was decreased by CovS inactivation in each of the acapsular strains (Fig. 5A). Similarly, we observed similar transcript level changes for directly CovR~P activated genes from MGAS2221 (i.e., decreased transcript levels following CovS inactivation) for the acapsular strains (Fig. 5B). Of these genes, only *braB*, which encodes a putative branched-chain amino acid permease protein (68), showed increased transcript levels in CovS-inactivated *emm28* but was unchanged in *emm87* and *emm89* ∆*covS* strains. Fig. 5C shows genes bound by CovR in MGAS2221 which did not have significant transcript level variation following CovS inactivation in the *emm*1 strain but did have transcript level variation following CovS inactivation in one or more of the acapsular strains. These included such important GAS virulence factor-encoding genes as the streptolysin S operon, *emm*, and *mga*. Finally, Fig. 5D shows genes whose transcript levels were impacted by CovS inactivation in one or more acapsular strains but are either absent or not bound by CovR in strain MGAS2221. These included the pilus operon, *enn* and *sof*, which encode cell-surface proteins, as well as *mf2*, which encodes an actively secreted DNase cotranscribed with *speC*, as well as *SpxA2*, which encodes a transcriptional regulator previously shown to impact expression of CovR-regulated genes through an unclear mechanism (69). Of the 69 core genes in the acapsular CovS transcriptome, 62 are present in MGAS2221 and 32 of these (52%) were CovR bound in MGAS2221. Taken together, we conclude that direct CovR regulation likely accounts for a majority of the acapsular CovS transcriptome and that CovS inactivation results in key distinction between encapsulated *emm1* strain MGAS2221 and acapsular GAS which will be further discussed below.

### CovR directly represses *ska* in acapsular and encapsulated strains yet CovS inactivation leads to differential impact on *ska* transcript levels

**Fig 6:**
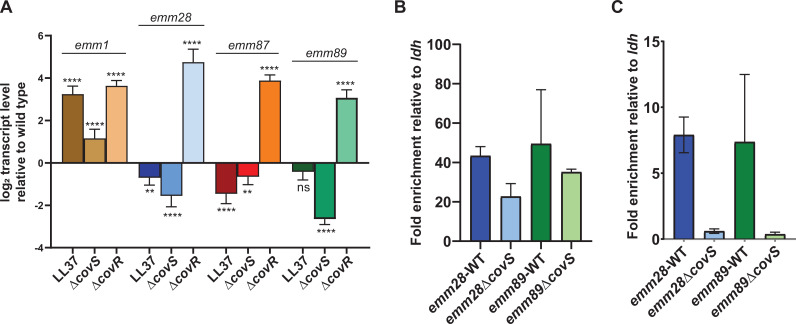
Impact of CovS inactivation on *ska* transcripts and *ska* promoter enrichment. (**A**) Taqman qRT-PCR analysis of *ska* transcript levels in response to LL37 and upon inactivation of CovS and CovR in *emm1*, *emm28*, *emm87,* and *emm89* GAS strains. Data shown are mean ± standard deviation from two biological replicates, with two technical replicates, done on two separate days. Unpaired Student *t*-test was used to determine statistical significance relative to wild type (**, *P* < 0.005; ****, *P* < 0.0001). Binding of CovR~P to promoter of (**B**) *ska* and (**C**) *2221–0159* was analyzed by ChIP-qPCR in *emm28* and *emm89* wild-type and CovS-inactivated strains. Data shown are mean ± standard deviation of at least two biological replicates in duplicate.

The first area of distinction we sought to investigate was the differential impact of CovS inactivation on *ska* transcript levels between the *emm*1 and acapsular strains that were also observed upon LL37 treatment (Fig. 6A). There are two major variants of the *ska* gene with the acapsular strains all harboring pattern 1 and *emm*1 harboring pattern 2 (70, 71). The *ska* promoters are approximately 90% conserved between the *emm*1 and acapsular strains with the major difference being a 21-bp insertion approximately 170 bps upstream of the ATG start codon and 120 bps from the major CovR binding site identified *in vitro* (72) (Fig. S3). To determine whether CovR directly repressed *ska* in the acapsular strains, we inactivated CovR in all three *emm* types as well as assayed for CovR-mediated enrichment at the *ska* promoter using chromatin immunoprecipitation followed by qPCR (ChIP-qPCR). Similar to observations in *emm*1, CovR inactivation increased *ska* transcript levels in each of the acapsular strains, and we detected DNA enrichment of the *ska* promoter by ChIP-qPCR for both *emm*28 and *emm*89 strains (Fig. 6B). The *ska* transcript level pattern in acapsular strains (i.e., CovR repression but CovS activation) resembles those described for both *speB* and *grab* and has been postulated to be due to increased binding of nonphosphorylated relative to phosphorylated CovR (7, 73). Thus, we next tested the hypothesis that lowering CovR~P levels via CovS inactivation would increase CovR-mediated enrichment at the *ska* promoter. Contrary to our hypothesis, CovS inactivation either decreased (*emm*28) or did not significantly change (*emm*89) CovR-mediated enrichment as measured using ChIP-qPCR. To ensure the validity of our ChIP experiment, we confirmed that CovS inactivation resulted in the expected decrease in CovR enrichment at the promoter of *2221_0159* for both *emm28* and *emm89* GAS (Fig. 6C) as has previously been published for *emm1* (17). These data suggest that CovR directly represses the *ska* promoter in both encapsulated *emm1* and acapsular strains but that factors beyond changes in CovR-DNA binding may account for the differential impact on *ska* transcript levels of altering CovR~P levels in encapsulated vs. acapsular strains.

### The *mga*/*emm* locus is upregulated in acapsular CovS-inactivated strains

**Fig 7:**
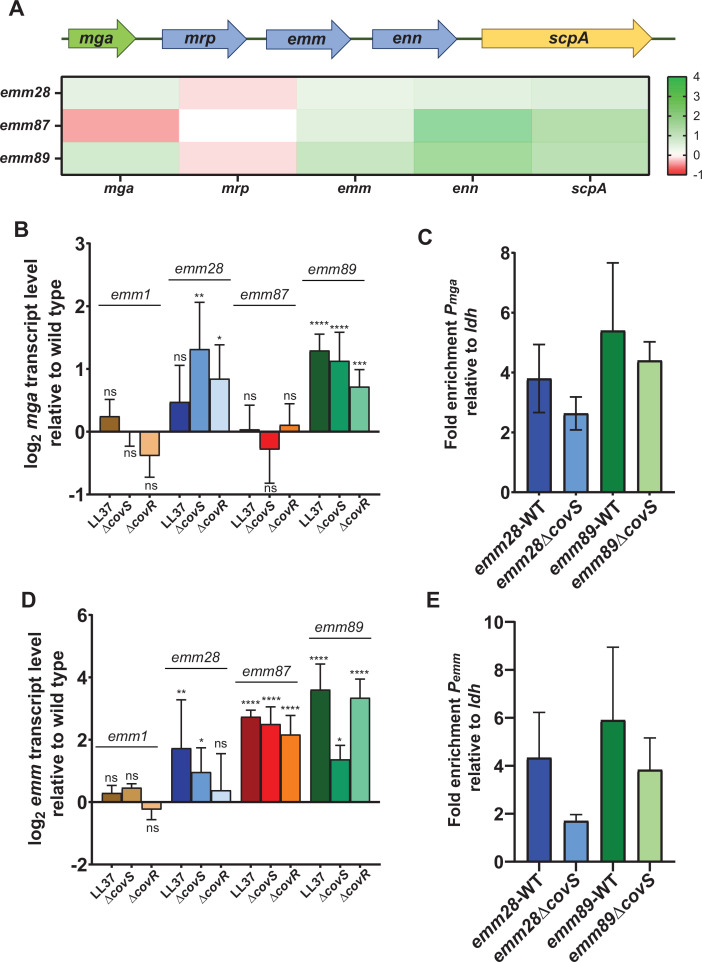
Impact of CovS inactivation on transcript levels and CovR-promoter binding for *mga* and *emm* in encapsulated *emm*1 and acapsular *emm*28, *emm*87, and *emm*89 strains. (**A**) Schematic representation of the *mga* region and heat map of transcript level changes observed in RNA-seq data for acapsular GAS strains. Genes upregulated upon CovS inactivation are indicated in green, and genes downregulated upon CovS inactivation are indicated in red. Transcript level changes for *mrp* were not statistically significant. Impact on transcript levels of (**B**) *mga* and (**D**) *emm* genes on treatment with LL37 and inactivation of CovS and CovR analyzed by Taqman qRT-PCR. Data shown are mean ± standard deviation from two biological replicates, with two technical replicates, done on two separate days. Unpaired Student *t*-test was used to determine statistical significance relative to wild type (ns, not significant; *, *P <* 0.05; **, *P <* 0.005; ***, *P <* 0.0005; ****, *P <* 0.0001). Fold enrichment of the (**C**) *mga* and (**E**) *emm* promoter relative to *ldh* in *emm28* and *emm89* wild-type and CovS-inactivated strains analyzed by ChIP-qPCR. Data shown are mean ± standard deviation of at least two biological replicates in duplicate.

RNA-seq revealed that CovS inactivation significantly increased the transcript levels of *emm* in all three acapsular strains and of *mga* in *emm*28 and *emm89* (Fig. 7A). These findings are in contrast to *emm*1 GAS in which CovR binds to the *emm* and *mga* promoters, but there is no impact of CovS inactivation on *mga* or *emm* transcript levels (17). The percent homology for the *mga* and *emm* promoter in the acapsular relative to *emm*1 GAS is ~60% and 75%, respectively, indicating potential variation in *cis*-regulatory elements between the acapsular and *emm*1 strains. We first confirmed our RNA-seq CovS inactivation data using qRT-PCR (Fig. 7B and D) and also found that treatment of all three acapsular strains with LL-37 resulted in similar alteration of *emm* and mga transcript levels as observed for CovS inactivation (Fig. 7B and D). The lack of an increase in *mga* transcripts upon CovS inactivation or LL-37 treatment in the *emm*87 strain might result from a 10nt deletion in the *mga* promoter of *emm87* that overlaps a putative CovR binding site as well as the -35 promoter element (Fig. S4). Inactivation of CovR significantly increased *emm* transcript levels for all three acapsular types and *mga* transcript levels for *emm*28 and *emm*89 (Fig. 7B and D). Given that *emm* is directly regulated by Mga, the increase in *emm,* but not *mga* in the *emm*87 strain, suggested that CovR may be directly regulating *emm* in addition to *mga*. Consistent with this hypothesis, we found that CovR binds the promoters of both *mga* and *emm* as measured by ChIP-qPCR in the *emm28* and *emm89* acapsular strains (Fig. 7C and E). Although we did observe a slight decrease in CovR-mediated DNA enrichment for the *emm*89 and *emm*28 strains at the *emm* and *mga* promoters following CovS inactivation, this difference was not statistically significant for either strain (Fig. 7C and E). Thus, these data indicate that CovR directly regulates both *emm* and *mga* in the acapsular strains and that CovS inactivation increases *emm* (all three strains) and *mga* (*emm*28 and *emm*89) transcript levels but is not clear whether a decrease in CovR at the *emm* and *mga* promoter drives this variation.

### CovS inactivation broadly increases transcript levels of cell-surface protein-encoding genes

**Fig 8:**
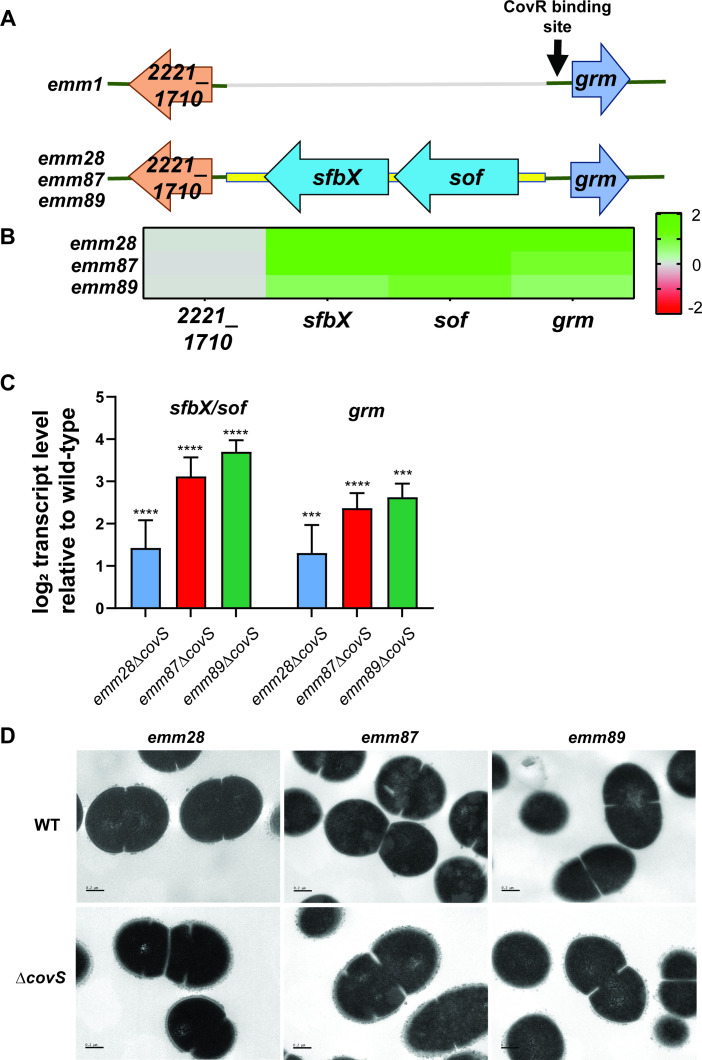
Effect of CovS inactivation on cell-surface protein-encoding genes and cell surface morphology. (**A**) Schematic representation of the differences in the *sof*/*sfbX*/*grm* region between encapsulated and acapsular GAS. (**B**) Transcript level alterations observed for the genes in RNA-seq data. Genes upregulated upon CovS inactivation are indicated in green, and genes downregulated upon CovS inactivation are indicated in red. (**C**) Taqman qRT-PCR validation of the transcript level changes observed in CovS-inactivated *emm28*, *emm87,* and *emm89* strains relative to wild type. Data shown are mean ± standard deviation from two biological replicates, with two technical replicates, done on two separate days. Unpaired Student *t*-test was used to determine statistical significance relative to wild type (***, *P <* 0.0005; ****, *P <* 0.0001). (**D**) Transmission electron micrographs showing the smooth cell surface of wild-type *emm28*, *emm87,* and *emm89* strains compared to the rough cell surface of their CovS-inactivated counterparts. Sections shown at a magnification of 40,000 with bars indicating 200 nm.

Our transcriptome data indicated that CovS inactivation led to consistent upregulation of genes encoding the cell-surface proteins serum opacity factor (Sof), which is a fibronectin-binding protein, and the fibrinogen-binding SfbX. These genes are encoded by a single operon and are absent in *emm1* GAS (Fig. 8A). Alignment of the *sof/sfbX* region showed significant homology for the upstream and downstream genes between the acapsular and *emm1* strains, suggesting either *en bloc* gene gain or gene loss leading to the observed arrangements. Divergently transcribed from *sof* is a gene previously called *grm* for gene regulated by Mga, which we have identified as directly regulated by CovR in *emm1* GAS (17, 74). The CovR binding site in the *grm* promoter is conserved between *emm1* GAS and the acapsular strains, which could potentially place the *sof/sfbX* promoter under CovR control. The transcript level patterns induced by CovS inactivation of *grm* and the *sof/sfbX* operon are highly similar in all three acapsular strains (Fig. 8B), suggesting that CovR may be directly regulating both. We confirmed that CovS inactivation consistently upregulated *sof* and *grm* by qRT-PCR (Fig. 8C). Overall, there were 10 putative cell-surface protein (i.e., those that contain LPXTG motifs) encoding genes consistently upregulated following CovS inactivation in the acapsular strains (*prtS*, *mac-1*, *sclA*, *sclB*, *fbpA*, *scpA*, *enn*, *emm*, *sfbX*, and *sof*). We had previously reported an increase in the abundance of several of these cell-surface proteins in acapsular *emm4*∆*covS* strains accompanied by a rough cell surface covered by protruding material (37). Thus, we used electron microscopy to evaluate how CovS inactivation impacted the cell surface of acapsular GAS and found a marked increase in cell-surface projections suggestive of increased amount of cell-surface proteins (Fig. 8D).

### CovS inactivation decreases transcript levels of pilus operon-encoding genes and adherence to human tonsillar epithelial cells in acapsular GAS

**Fig 9:**
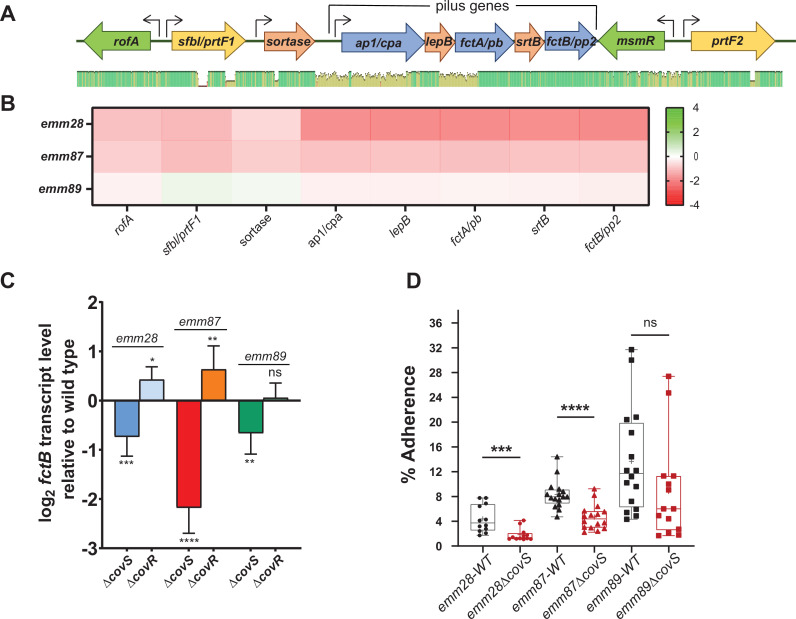
Impact of CovS inactivation on transcription of GAS pilus operon and adherence to primary tonsillar epithelial cells. (**A**) Schematic representation of the FCT region including the pilus operon and the variation in the pilus operon genes among *emm28*, *emm87,* and *emm89* strains. Promoters are indicated by black arrows. The bar below the schematic indicates identity. Residues that are identical in all three strains are denoted by green bars, and residues that differ are indicated by brown bars. (**B**) Transcript level alterations observed for FCT genes in RNA-seq data. Genes upregulated upon CovS inactivation are indicated in green, and genes downregulated upon CovS inactivation are indicated in red. (**C**) Analysis of the impact of inactivation of CovS and CovR in *emm28*, *emm87,* and *emm89* strains on transcription of the pilus genes by Taqman qRT-PCR relative to wild type. Data shown are mean ± standard deviation from two biological replicates, with two technical replicates, done on two separate days. Unpaired Student *t*-test was used to determine statistical significance relative to wild type (*, *P <* 0.05; **, *P <* 0.005; ***, *P <* 0.0005; ****, *P <* 0.0001). (**D**) Adherence of wild-type (black symbols) and CovS-inactivated (red symbols) *emm28*, *emm87,* and *emm89* strains to tonsillar epithelial cells. Statistical significance was determined by Mann-Whitney test (ns, not significant; ***, *P <* 0.0005; ****, *P <* 0.0001).

The finding that genes of the pilus operon are downregulated by CovS inactivation in acapsular strains was intriguing given that pilus-related genes have not been observed as being impacted by CovS in encapsulated GAS, such as *emm1* or *emm3*. The arrangement of the fibronectin and collagen-binding proteins and trypsin-resistant antigens (FCT) region from *emm28*, *emm87*, and *emm89* GAS (FCT4 variant) along with RNA-seq transcript level data is shown in Fig. 9A and B. We identified significant genetic heterogeneity among the FCT region for the acapsular strains except for the last two genes of the pilus operon, *srtB* and *fctB*, which encode a sortase and minor pilus subunit, respectively (Fig. 9A). Thus, we used *fctB* to measure pilus gene transcript levels, and consistent with the RNA-seq data, qRT-PCR showed significantly lower *fctB* transcript levels in all three *emm* types following CovS inactivation (Fig. 9C). Conversely, CovR inactivation either increased *fctB* transcript levels (*emm28* and *emm87*) or caused no change (*emm89*) (Fig. 9C). The divergent impact of CovR vs. CovS inactivation on pilus operon transcript levels suggested that the impact of CovS inactivation on FCT region genes might be indirect. Consistent with this hypothesis, we did not observe significant CovR-mediated DNA enrichment of any of the promoters within the FCT locus. The GAS pilus is important for GAS adherence to host cells, including tonsillar epithelium (75, 76). Thus, we analyzed the impact of CovS inactivation on the adherence of *emm28*, *emm87,* and *emm89* strains to primary tonsillar epithelial cells (HTEpiC). For *emm28* and *emm87*, CovS inactivation significantly reduced adherence (Fig. 9D). For *emm89*, the observed reduction in adherence between wild-type and the ∆*covS* strain was not statistically significant due to large intrastrain variation (Fig. 9D). These results are in accord with the relative magnitude of transcriptional changes that were observed for the FCT locus genes upon CovS inactivation (Fig. 9B).

## DISCUSSION

The recent recognition that about 30% of GAS strains lack hyaluronic acid capsule was surprising given that capsule has long been identified as central to many critical aspects of GAS pathogenesis such as inhibition of phagocytosis (31, 36, 77). Moreover, immune avoidance resultant from increased capsule production arising from abrogated CovR repression is considered a major contributor to CovS-dependent GAS hypervirulence (10, 78). In turn, this upregulation of capsule has been suggested to mask critical cell-surface adherence molecules necessary for the initial stages of GAS host-pathogen interaction, which provides a potential mechanism for the general lack of transmission of CovS-inactivated strains (13). Herein, we extended understanding of CovS inactivation to common, but relatively understudied, acapsular GAS *emm* types through a combination of genomic, transcriptomic, and functional studies that challenge current paradigms regarding the dynamics between CovS inactivation and capsule production and the impact of CovS inactivation on GAS pathophysiology.

A key finding of our work was that acapsular *emm* types causing invasive infections have similar rates of CovRS variation, including complete inactivation of CovS, relative to encapsulated strains. Relative to encapsulated GAS, one might have expected to observe less CovRS variation for acapsular strains given the lack of advantage induced by hyperproduction of capsule. These findings suggest that CovRS variation in invasive, acapsular GAS still confers a fitness advantage during host-pathogen interaction, probably as a result of the increased production of a broad array of cell surface proteins and secreted toxins and are consistent with previous studies of CovS inactivation in *emm4* and *emm87* backgrounds (37, 52). It was recently identified that the *M* protein from *emm*87 is able to bind and confer resistance to the important human antimicrobial peptide LL-37 (79) and improves survival in human blood (80) such that the upregulation of *emm* induced in acapsular strains following CovS inactivation may have similar immune avoidance properties as that conveyed by augmented capsule. Additionally, CovS inactivation significantly increased *mga* transcript levels which likely further increased the expression of Mga-activated genes encoding such key cell-surface proteins as *M* protein, C5a peptidase, and SclA (74). Interestingly, we identified direct CovR binding to the *mga* promoter in the acapsular strains as well as in *emm*1 GAS, but only in the acapsular strains did CovS inactivation increase *mga* transcript levels (17). Of note, hyperphosphorylation of CovR by abrogating CovS phosphatase activity diminished *mga* transcript levels in both *emm*1 and *emm*3 GAS consistent with CovR having some activity at the *mga* promoter even in encapsulated strains (6). Given the marked difference in *mga* promoter composition between *emm*1/3 and the acapsular strains studied herein, we hypothesize that variance in *cis*-regulatory elements may underlie differential impacts of varying CovR~P levels on *mga* transcript levels.

A second important aspect of our study was our identification of similar low rates of genetic clustering of invasive, *covRS*-mutated GAS strains irrespective of capsule status. These data are consistent with recent CDC publications that studied genetic clustering of either all invasive GAS (i.e., not just strains with CovRS mutations) (81) or CovS-inactivated strains in Colorado (21) which did not identify any particular predilection for clustering based on capsule status. Previous genomic studies have found that most CovRS-altered strains cause a single invasive infection suggesting limited transmissibility (19 - 52 - 52). For *emm*1 GAS, it was shown that hyperproduction of capsule reduces adherence to epithelial cells, thus rendering a fitness cost for CovRS inactivation (13). If such a hypothesis is correct, then one might have expected to observe clusters of invasive infections due to genetically related acapsular strains with the same CovRS variation. Our finding of increased multiplication and decreased adherence following CovS inactivation is consistent with previous data from members of our group studying CovS inactivation in a different *emm87* strain (52). The identification that CovS inactivation resulted in a decrease in pilus operon transcript levels provides a mechanism for the reduced adherence observed in both studies as well as a parallel pathway for limiting transmission of acapsular GAS analogous to augmented capsule production in encapsulated GAS. The precise role of the pilus in GAS pathogenesis has been difficult to clearly delineate due to variation in both pilus content and pilus expression (75, 82). An overall theme, however, has emerged where pilus production positively impacts the establishment of GAS colonization at mucosal surfaces through augmented attachment to epithelial cells but negatively impacts invasive GAS disease by facilitating neutrophil-mediated killing (76, 83). Thus, the decreased pilus transcript levels resulting from CovS inactivation may positively impact acapsular GAS interaction with human immune components but decrease the subsequent transmission of CovS-inactivated isolates. Alternative or additional explanations for the lack of transmission of CovS-inactivated strains have also been proposed (18) including poor growth under nutrient-limited conditions such as human saliva (12).

The consistent decrease in *ska* transcript levels following CovS inactivation in the acapsular strains stands in stark contrast to the increase in *ska* expression observed in CovS-mutated, encapsulated GAS (6, 11, 46). Given that streptokinase converts human plasminogen to plasmin which subsequently degrades extracellular matrix components, an increase in SKA activity is considered a key mechanism by which CovS inactivation augments GAS invasiveness (16). Bernard et al. previously noted that inactivation of the regulator of CovR (RocA), which diminishes CovR~P levels, also reduced *ska* transcript and SKA activity in *emm*28 strains, although they were unable to determine a mechanism (84). Herein, we show that *ska* transcript levels are reduced by CovS inactivation in all three studied acapsular *emm* types but that CovR remains a direct repressor of *ska* expression in *emm1* and acapsular GAS. Thus, for acapsular GAS, the *ska* gene is regulated in a fashion similar to *grab*, which is directly repressed by CovR yet shows reduced transcript levels following CovS inactivation. Given that we did not observe increased CovR-mediated enrichment of *ska* promoter DNA in CovS-inactivated strains, the mechanism of the varied impact of CovS inactivation on *ska* transcript levels in encapsulated vs. acapsular strains remains enigmatic. Additionally, it is not clear what evolutionary advantage of decreased SKA activity would be engendered specifically in acapsular CovS-inactivated strains, but further study of this difference may shed new light on GAS pathogenesis.

Our finding that the *emm*28 strain had lower baseline CovR~P levels relative to *emm*87, *emm*89, and *emm*1 strains adds to growing information regarding the apparently atypical nature of the CovRS system in this particular *emm* type (44). Bernard et al. noted that *emm*28 strains have an unusually high number of RocA polymorphisms relative to other *emm* types and demonstrated a dramatic impact of RocA on the *emm*28 transcriptome (85). Moreover, an analysis of over 2,000 *emm*28 strains identified numerous CovRS polymorphisms that were associated with a markedly altered transcriptome and increased virulence in a mouse necrotizing fasciitis model (44). In concert with the Bernard et al. study, we found that *emm*28 strains had the highest rates of CovRS polymorphisms (~35%) for invasive strains collected by the CDC and that the *emm*28 CovS transcriptome was larger than either the *emm*87 or the *emm*89 strains. This finding is somewhat counterintuitive given that the impact of CovS inactivation on GAS gene expression is thought to occur by decreasing CovR~P levels (4) and that the decrease in CovR~P following CovS inactivation was least for the *emm*28 strain. Indeed, for CovR~P-repressed genes such as *prtS* and *sclA*, the transcript level increases were lowest following CovS inactivation in *emm28* compared with *emm*87 and *emm*89. However, we identified large tracts of downregulated genes in the *emm28*Δ*covS* strain which were present in gene regions unique to *emm*28, including RD2. RD2 was likely acquired from GBS, contains known and putative transcriptional regulators, and has previously been shown to alter the GAS transcriptome (86). In GBS, CovR serves to silence expression of recently acquired DNA (87), and we hypothesize that CovRS influence on RD2 expression may contribute, in part, to the magnitude of the CovS transcriptome in GAS, much of which is likely indirect in nature given the large number of contiguous genes with similar transcript patterns despite being in distinct operons.

In conclusion, we present herein the most comprehensive analysis to date of CovS inactivation in acapsular GAS. We found that CovRS variation is common among acapsular GAS but that similar to encapsulated GAS, transmission of CovRS-mutated, acapsular GAS appears to be relatively rare, perhaps due to unique downregulation of pilus components induced by CovS inactivation. Our data suggest that CovS inactivation likely impacts virulence of acapsular strains through different mechanisms given the unique differences in gene regulation observed in acapsular ∆*covS* GAS strains. Further study of how acapsular GAS achieves hypervirulence in the absence of capsule may provide novel insights into GAS pathogenesis.

## MATERIALS AND METHODS

### Genome analysis

Paired-end short-read data were retrieved from NCBI using the sra-toolkit-v3.0.0 fasterq-dump script. Short-reads were then aligned to reference capsular (RefSeq #: *emm1*: NZ_CP043530.1, *emm3*: NZ_CP033815.1, and *emm12*: NZ_CP009612.1) and acapsular (*emm28*: NC_007296.2, *emm87*: NZ_CP007560.1, and *emm89*: NZ_CP013839.1) GAS complete genomes using the snippy-v4.6.0 (Seemann T, snippy, GitHub: https://github.com/tseemann/snippy) core SNP phylogeny pipeline. A core SNP alignment was then used as input to Gubbins-v3.2.1 (88), which can estimate regions of high recombination through measuring variance in SNP densities. A core SNP inferred maximum-likelihood phylogeny was built downstream with IQTree-v2.0.3 (89) as the Gubbins tree-model option using a generalized-time reversible nucleotide substitution model and gamma distribution to model rate heterogeneity. Branch values were tested using UFBoot (*n* = 1,000) and SH-Test (*n* = 1,000)

Variants of *covR* and *covS* from the Snippy-core pipeline were extracted and merged using bcftools-1.15. The R library VariantAnnotation-1.42.1 was used to annotate and predict coding changes from reference genomes. A custom R script was adapted to annotate and extract variants using the above R library (S Selvalakshmi, VariantAnnotator, GitHub: https://github.com/Selvalakshmi27/VariantAnnotator). The pipeline uses merged vcf file output from bcftools and a reference genbank file as inputs. In case of a gff3 file as an input reference file, agat_convert_sp_gxf2gxf.pl script from agat-1.0.0 was used to index and sort gff3 files before using the VariantAnnotator pipeline. The output is a csv file containing the predicted coding changes of the variants along with the consequences of the change (synonymous/nonsynonymous). Nonsynonymous *covR* and *covS* mutations that occurred in two or greater isolates of each respective emm type were mapped onto respective phylogenies and visualized using iTOL-v6 (90).

### Bacterial strains, growth conditions, and mutant generation

Strains were grown in a nutrient-rich standard laboratory medium [Todd-Hewitt broth with 0.2% yeast extract (THY)] at 37°C with 5% CO_2_. Phenotypic characterization was performed following growth on sheep blood agar plates. Nonpolar insertional mutagenesis with a spectinomycin resistance cassette was employed to obtain isogenic *covS* and *covR* mutants in representative strain of *emm*28, *emm*87, and *emm*89 GAS as described previously (Table 1) (39, 43). The *emm*1 MGAS2221 and its isogenic ∆*covS* and ∆*covR* mutants has been previously described (12).

### Detection of CovR phosphorylation status

Recombinant CovR was produced and phosphorylated as described previously (6). GAS lysates were prepared from mid-exponential cultures, separated on 10% Phos-Tag SDS-polyacrylamide gels, and unphosphorylated/phosphorylated CovR proteins were detected by using a polyclonal anti-CovR antibody as described previously (6) using an Odyssey imaging system.

### Transcript level analysis and RNA-seq

RNA was purified from various GAS strains grown to mid-exponential phase (OD_600_~0.5), and TaqMan real-time qRT-PCR was performed as described previously (6, 91, 92) (primers and probes listed in Table S3). All strains were collected in duplicate on at least two separate occasions and analyzed in duplicate.

For RNA-seq analysis, strains were grown on two different days to mid-exponential phase in THY, and RNA was isolated as for TaqMan qRT-PCR. RNA library preparation followed by RNA-seq was performed by the MD Anderson core genome center to generate raw paired-end RNA reads (75 × 2). RNA-seq quality control was done on untrimmed reads using fastqc-v0.11.8. Untrimmed QC’d reads were aligned to each respective complete assembly [NC_007296 (*Streptococcus pyogenes* MGAS6180) for *emm28* strain TSPY902, NZ_CP007560 (*S. pyogenes* NGAS743) for *emm87* strains TSPY1057, and CP013840.1 (*S. pyogenes* MGAS27061) for *emm89* strain MSPY1] using the STAR aligner (93). The HTSeq python package (94) was used for enumerating read counts of uniquely mapped reads using the *Aligned.sortedByCoord.out.bam STAR output file along with a reference gtf file. RNA-seq analysis was performed as described previously (37, 91, 95). Briefly, differential expression between wild-type and CovS mutant strains was performed using the DESeq2 package-v1.30.1 (96). Read counts from HTSeq were normalized by estimating size factors using the median ratio method employed in estimateSizeFactors function. Genes with less than one per million normalized reads mapped for all samples were excluded from downstream analyses. The DESeq2::nbinomWaldTest function is used to test for differential expression, employing a Wald test that tests the significance of coefficients from a negative binomial generalized linear model fit to each gene and is corrected for multiple comparisons using a false discovery rate. A fold change of 1.5× with an alpha parameter = 0.05 was used for these calculations. A principal component analysis was used to visualize the clustering of expression profiles by sample.

### Growth in human blood

Lancefield assays were performed as previously described (97) under a protocol approved by the Committee for the Protection of Human Subjects at the McGovern Medical School at UTHealth Houston. Blood samples from four healthy, nonimmune, adult donors were used for each strain, and assay was performed in triplicate. Strains were grown to mid-exponential phase (OD_600_ ~ 0.5) in THY, and cells were pelleted and resuspended in phosphate-buffered saline (PBS). To assay growth, ~100 CFUs of each strain were added to 300 µL of blood, incubated for 3 h, and dilutions plated on blood agar plates for enumeration. Multiplication factors were calculated by dividing the number of CFU per milliliter after 3 h of incubation by the initial inoculum.

### Chromatin immunoprecipitation and SYBR qRT-PCR

Chromatin immunoprecipitation was performed for *emm28* and *emm89* strains as described previously (17, 92). Briefly, GAS strains were grown in THY medium to mid-exponential phase, proteins were cross-linked to DNA, and cells were subsequently harvested. Cell pellets were resuspended in lysis buffer, sonicated in a Diagenode Bioruptor Plus machine, and CovR-bound DNA fragments were immunoprecipitated using a polyclonal antibody directed against the N-terminal domain of CovR (CovR_ND_) (17). Both ChIP and input DNA were purified. Enrichment of selected promoters in ChIP samples derived from wild-type and CovS-inactivated strains relative to input DNA was assessed by SYBR qRT-PCR as described previously (17) using primers listed in Table S3. The fold enrichment of promoters of interest was normalized to that of the *ldh* promoter region (not CovR regulated). Measurements were done in duplicate on at least three biological (*n* = 3) samples.

### Microscopy

The cell surface morphology of wild-type and CovS-inactivated strains was analyzed using transmission electron microscopy. Cells were prepared, fixed, and sectioned as described previously (37). Images were obtained using a Jeol 1200 transmission electron microscope with a Gatan digital camera.

### Adherence assay

Adherence of wild-type and CovS-inactivated strains to human tonsillar epithelial cells was assayed as described previously (52). Briefly, mid-exponential GAS cells (OD_600_ ~ 0.5) were washed, resuspended in PBS, and used to infect wells seeded with 0.5–1 × 10^6^ epithelial cells at an MOI of 10 in 4 technical replicate wells. Plates were incubated at 37°C for 90 min, washed, and lysed with Trypsin. Dilutions were plated on blood agar plates to enumerate, and percent adherence was calculated based on CFUs recovered on blood agar plates. The experiment was repeated on four different days.

## Data Availability

The RNAseq data has been submitted to the GEO depository (GSE230158) and will be publicly available.

## SUPPLEMENTAL MATERIAL

Figure S1 msystems.00227-23-s0001.tif Occurrence and genetic clustering of *covRS* mutations.

Figure S2 msystems.00227-23-s0002.eps Principal Component Analysis for RNA-seq.

Figure S3 msystems.00227-23-s0003.eps Variation in the *ska* promoter.

Figure S4 msystems.00227-23-s0004.eps Variation in the *mga* promoter.

Table S1 msystems.00227-23-s0005.xlsx Occurrence and distribution of CovRS mutations in invasive GAS strains.

Table S2 msystems.00227-23-s0006.xlsx RNA-seq data for analysis of wild-type and CovS-inactivated strains.

Table S3 msystems.00227-23-s0007.xlsx Primers and probes used in the study.

Supplemental Legends msystems.00227-23-s0008.docx Legends for supplemental tables and figures.

ASM does not own the copyrights to Supplemental Material that may be linked to, or accessed through, an article. The authors have granted ASM a non-exclusive, world-wide license to publish the Supplemental Material files. Please contact the corresponding author directly for reuse.
