# Supplementary figures and images for: Identification of distinct impacts of CovS inactivation on the transcriptome of acapsular group A streptococci
Source: mSystems. 2023 Jun 26;8(4):e00227-23. doi: 10.1128/msystems.00227-23 (PMC10470059; doi:10.1128/msystems.00227-23)

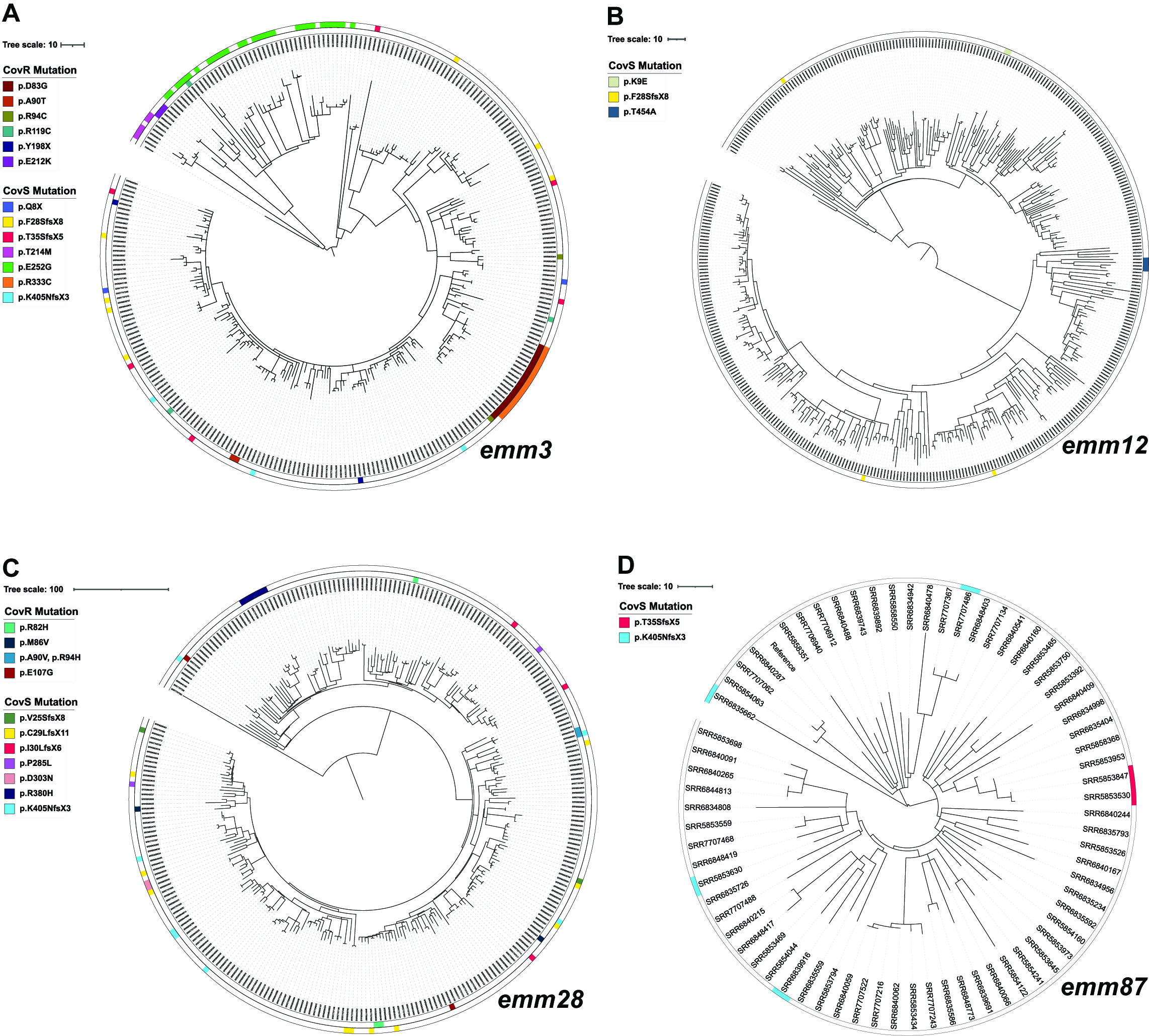

Supplement: Figure S1 — Occurrence and genetic clustering of covRS mutations. [file msystems.00227-23-s0001.tif]
