# Supplementary material for: Identification of distinct impacts of CovS inactivation on the transcriptome of acapsular group A streptococci
Source: mSystems. 2023 Jun 26;8(4):e00227-23. doi: 10.1128/msystems.00227-23 (PMC10470059; doi:10.1128/msystems.00227-23)
Supplement: Supplemental Legends — Legends for supplemental tables and figures. [file msystems.00227-23-s0008.docx]

**SUPPLEMENTARY MATERIAL**

**Supplementary Tables**

Table S1. Distribution of *covR* and *covS* mutations identified in GAS strains.

Table S2. List of differentially expressed genes in acapsular *emm28*, *emm87* and *emm89* strains upon CovS inactivation.

Table S3. Primers and probes used in this study.

**Supplementary Figure Legends**

**Supplementary Figure S1. Occurrence and genetic clustering of *covRS* mutations.** A recombination-free, core genome alignment inferred maximum-likelihood phylogenetic tree was created from alignment of (A) 282 *emm3*, (B) 404 *emm12*, (C) 281 *emm28*, and (D) 69 *emm87* strains. The occurrence of mutations in *covR* and *covS* are indicated on the inner and outer circles respectively and color coordinated for each specific mutation as detailed in the legend.

**Supplementary Figure S2. Principal Component Analysis for RNA-seq.** Wild type and CovS inactivated mutants of *emm28*, *emm87* and *emm89* show distinct transcriptomes. Each strain was analyzed in duplicate.

**Supplementary Figure S3**. **Variation in the *ska* promoter.** Alignment of the promoter region of *ska* from *emm1*, *emm28*, *emm87* and *emm89*. A 21bp region absent in acapsular strains and the CovR binding site (as determined by ChIP-seq) are indicated.

**Supplementary Figure S4**. **Variation in the *mga* promoter.** Alignment comparing the promoter region of *mga* from *emm28/89* to that of *emm87*. The emm87 promoter has a 10nt deletion that overlaps with the -35 promoter element and a putative CovR binding site.
